# Antifreeze Protein for Freeze–Thaw Durability Enhancement of Cement Mortar: Effects and Action Analysis

**DOI:** 10.3390/ma19101997

**Published:** 2026-05-12

**Authors:** Qiyu Zhang, Jingwei Gong, Miaomiao Gong

**Affiliations:** 1 College of Hydraulic and Civil Engineering, Xinjiang Agricultural University, Urumqi 830052, China; 2 Xinjiang Key Laboratory of Hydraulic Engineering Security and Water Disasters Prevention, Urumqi 830052, China

**Keywords:** cement mortar, antifreeze protein, ice-crystal morphology, pore size distribution, frost-heaving stress

## Abstract

Enhancing the freeze–thaw resistance of cement-based materials in a green and efficient manner is crucial for hydraulic structures in cold regions. This study investigated the effects of soybean antifreeze protein (AFP) on the freeze–thaw durability of cement mortar through mechanical testing, low-temperature microscopy, NMR analysis, and frost-heaving stress monitoring. The results show that AFP improves freeze–thaw durability, with 0.5% dosage outperforming 1.0%. Relative to the control, the relative ice content at −20 °C decreased from 62.81% to 40.01%, and frost-heaving stress declined from 321.15 kPa to 123.04 kPa. Microscopy and pore structure analyses revealed that AFP transforms ice crystals from needle-like to fine granular forms, inhibiting ordered growth and retarding pore coarsening. A frost-heaving stress model based on the Gibbs–Thomson effect and ice-crystal fractal characteristics indicated that AFP suppresses stress development by reducing effective ice formation, weakening stress transfer, and increasing ice-crystal boundary complexity. This study offers insights for developing green antifreeze admixtures for cement-based materials in cold regions.

## 1. Introduction

Concrete serves as a core material for infrastructure construction across transportation, building, hydraulic engineering, and energy sectors due to its excellent mechanical properties, wide availability of raw materials, and high cost-effectiveness. Nevertheless, the deterioration of freeze–thaw durability during service in cold regions constitutes a critical bottleneck that restricts the safe operation of such infrastructure [1,2,3]. The cyclic liquid–solid phase transition of the pore solution induces volumetric expansion and progressive stress accumulation, ultimately resulting in microcrack propagation, pore structure coarsening, and the degradation of mechanical performance [4,5,6,7]. In this context, the development of green and efficient antifreeze modification strategies is of substantial engineering and scientific significance.

Antifreeze proteins (AFPs) constitute a class of naturally occurring bioactive macromolecules derived from a variety of organisms, including plants, fish, insects, and microorganisms [8,9,10,11,12]. These proteins exhibit distinctive capabilities in inhibiting ice-crystal growth, modulating ice-crystal morphology, and suppressing ice recrystallization [13,14,15,16,17]. In contrast to conventional antifreeze agents, which mitigate freezing damage primarily by depressing the freezing point of the system, AFPs function predominantly through selective recognition and adsorption onto specific ice-crystal surfaces. This adsorption alters the growth kinetics and morphological evolution at the ice–water interface, thereby impeding the continuous propagation of ice crystals along preferred crystallographic planes [18,19,20]. Owing to this mechanism of direct intervention in the critical microscopic processes of the liquid–solid phase transition, AFPs have demonstrated considerable application potential in diverse fields, including food preservation, cryopreservation of biological specimens, anti-icing surfaces, and oil and gas transportation [21,22,23,24]. From a mechanistic standpoint, AFPs offer a novel strategy for enhancing the frost resistance of cement-based materials that is fundamentally distinct from conventional approaches reliant on freezing-point depression or straightforward pore structure refinement. By influencing the nucleation and growth behavior of ice crystals within the pore solution, AFPs can attenuate the fundamental basis of freezing damage at its source, thereby exhibiting substantial research significance and practical value.

To date, research on antifreeze proteins has predominantly centered on biocryopreservation, food engineering, and anti-icing functional materials, with their mechanisms of ice-growth inhibition, thermal hysteresis, and interfacial adsorption being relatively well established. In recent years, a limited number of investigations have attempted to introduce biomimetic antifreeze strategies into asphalt, protective coatings, and cement-based material systems, thereby providing preliminary evidence for the beneficial role of bio-derived antifreeze components in improving low-temperature frost resistance [25,26,27]. Nevertheless, the application of AFPs in cement-based materials remains in its nascent stages, and existing studies have largely focused on the characterization and evaluation of macroscopic freeze resistance. Notably, the cross-scale pathway linking ice-crystal morphology regulation to the enhancement of macroscopic durability has yet to be fully elucidated through experimental investigation. In particular, the intrinsic relationships and transfer mechanisms among key processes—including variations in relative ice content, pore-structure response, and the accumulation of frost-heaving stress—remain insufficiently clarified.

On this basis, ordinary Portland cement mortar was selected as the research object, and soybean-derived antifreeze protein (AFP) was incorporated at dosages of 0%, 0.5%, and 1.0% by mass. Low-temperature microscopy, nuclear magnetic resonance (NMR) spectroscopy, and frost-heaving stress monitoring were employed to systematically investigate the influence of AFP on ice-crystal morphology, relative ice content, pore structure evolution, and freeze–thaw durability. Based on the morphological characterization of ice crystals, the measured relative ice content, and the acquired frost-heaving stress data, an analytical model for frost-heaving stress was developed that accounts for the fractal characteristics of ice crystals. Using this model, the underlying causes of the variation in frost-heaving stress induced by AFP incorporation were further discussed. The findings of this study provide experimental insights into the effects of AFP on the freezing behavior and frost resistance of cement mortar, and offer a valuable reference for the development and engineering application of environmentally friendly frost-resistant mortar in cold-region environments.

## 2. Materials and Methods

### 2.1. Materials

#### 2.1.1. Cement

P·O 42.5 ordinary Portland cement (Tianshan Cement Plant) (Urumqi, China) was used. Its physical, mechanical, and chemical properties comply with GB 175-2023 [28] Its main physical properties and chemical composition are listed in Table 1 and Table 2, respectively.

#### 2.1.2. Soybean Antifreeze Protein

The soybean antifreeze protein (AFP) used in this study was provided by Sichuan Chuankuo Biotechnology Co., Ltd. (Chengdu, China). The main amino acid components are shown in Table 3.

#### 2.1.3. Fine Aggregate and Mixing Water

ISO standard sand was used as fine aggregate. Laboratory-prepared distilled water was employed for mixing, and its quality complied with the requirements specified in Specifications and Test Methods for Water Used in Analytical Laboratories (GB/T 6682-2008) [29].

### 2.2. Specimen Preparation and Test Methods

#### Specimen Preparation

**Figure 1:**
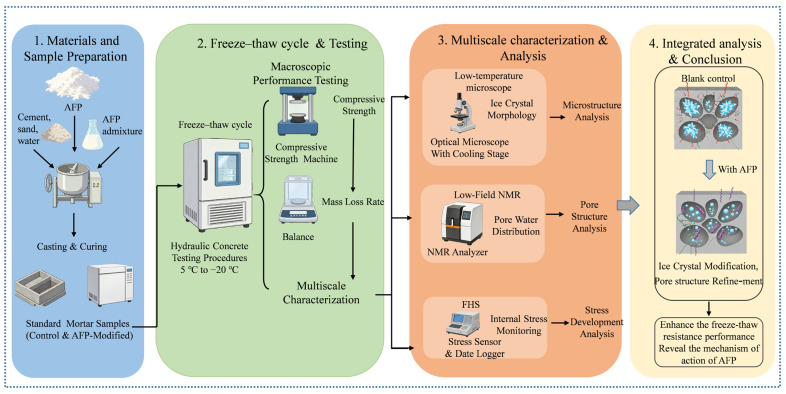
Technical route of this study.

The water-to-solid ratio was fixed at 0.50, and AFP dosages of 0%, 0.5%, and 1.0% by cement mass were used. The mixtures were denoted as KB, Pr0.5%, and Pr1%, respectively, where KB represents the control group without AFP. The detailed compositions are listed in Table 4. For clarity, prismatic and cylindrical specimens are identified in Table 4 by the suffixes -p and -c, respectively. Cement mortar specimens were prepared in accordance with GB/T 17671-2021 [30]. After casting, all specimens were demolded after 24 h and cured in water under standard curing conditions for 7 days prior to testing. Prismatic specimens were used for freeze–thaw cycling, compressive strength, mass loss, and NMR measurements because this geometry conforms to the standard testing procedure for cement mortar and facilitates the evaluation of macroscopic durability degradation. Cylindrical specimens were used for frost-heaving stress monitoring because the customized apparatus required a restrained cylindrical geometry to ensure uniform confinement and reliable embedding of the vibrating-wire strain gauge. Freeze–thaw cycling was conducted in accordance with SL/T 352-2020 [31] over a temperature range of −18 °C to 5 °C, with a cycle duration of 3 h. Compressive strength was measured every 25 cycles, while mass was recorded every 10 cycles during the first 60 cycles and every 25 cycles thereafter. The technical route is illustrated in Figure 1.

### 2.3. Test Indices

#### 2.3.1. Compressive Strength Test

After the specimen reached the 7-day curing age or completed the designated number of freeze–thaw cycles, they were promptly removed from the chamber, and surface moisture was gently wiped off with a damp cloth. Flexural and compressive strength tests were subsequently performed in accordance with the Method of Testing Cement—Determination of Strength (ISO Method) (GB/T 17671-2021). Three replicate specimens were prepared for each group, and the average value was reported as the test result. The error bars shown in the corresponding figures represent the standard deviation of the three replicate measurements. The compressive strength was calculated using Equation (1), with a loaded area of 1600 mm^2^. The relative compressive strength was determined according to Equation (2).

(1) $$R_{c}=\frac{F_{c}}{A}$$

where *R_c_* is the compressive strength (MPa), *F_c_* is the maximum load at failure (N), and *A* is the loaded area (mm^2^).

(2) $$K_{f}=\frac{f_{t}}{f_{0}}\times 100\%$$

where *K_f_* is the relative compressive strength (%), *f_t_* is the compressive strength of the specimen after *t* freeze–thaw cycles (MPa), and *f*_0_ is the initial compressive strength of the specimen (MPa).

#### 2.3.2. Mass Loss Test

Upon reaching the specified curing age, the mass of each specimen was determined using an electronic balance. Three replicate specimens were prepared for each group, and the average value was adopted as the final result. The error bars shown in the corresponding figures represent the standard deviation of the three replicate measurements. The mass loss ratio was calculated according to Equation (3). A specimen was deemed to have failed when the mass loss ratio attained 5%.

(3) $$\Delta W_{n}=\frac{W_{0i}-W_{ni}}{W_{ni}}\times 100\%$$

where W_n*i*_ is the mass loss ratio of the specimen after *n* freeze–thaw cycles (%), W_0*i*_ is the initial mass of the specimen (g), and *W*_n_ is the mass of the specimen after *n* freeze–thaw cycles (g).

#### 2.3.3. Frost-Heaving Stress Test

**Figure 2:**
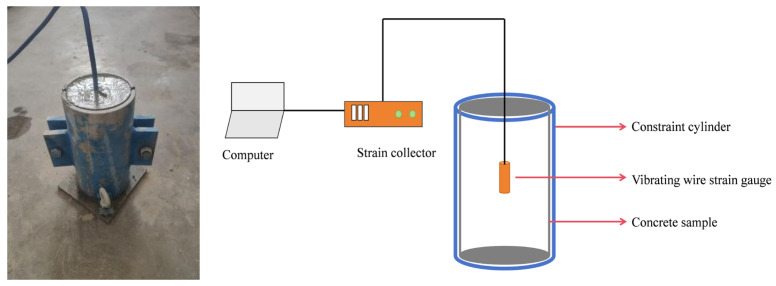
Schematic diagram of the FHS testing apparatus.

Cylindrical specimens were water-cured for 7 days and subsequently employed for frost-heaving stress (FHS) testing. To directly evaluate the effectiveness of AFP in mitigating FHS, a customized apparatus was designed to measure the overall internal FHS developed within the specimens. The apparatus comprised a confining steel cylinder, a vibrating-wire strain gauge, and a strain data acquisition system. The steel confining cylinder served to restrain specimen deformation, while the internal frost-induced expansive strain of the cement mortar was monitored by the vibrating-wire strain gauge coupled with the strain collector. The vibrating-wire strain gauge was embedded directly within the cement mortar matrix. Considering the anisotropic nature of the specimen, the arrangement of both the confining steel cylinder and the cement mortar specimen was configured as depicted in Figure 2. The FHS measurement procedure is outlined as follows:

(1) The initial temperature of the specimen was stabilized at 20 °C, while the confining cylinder was pre-cooled and maintained at −20 °C for 24 h.

(2) Following assembly, the specimen was subjected to freezing at −20 °C. Owing to the rigid restraint imposed by the confining cylinder, specimen deformation was effectively constrained, and the strain recorded by the gauge originated exclusively from the specimen response.

(3) Strain data were recorded continuously, and the FHS was calculated according to Equation (4):

(4) $$\sigma =E_{d}\cdot \varepsilon$$

where *σ* is the frost-heaving stress (kPa), *ε* is the internal strain (με), and *E_d_* is the dynamic elastic modulus of concrete (GPa).

The dynamic elastic modulus was calculated according to Equation (5) [32]:

(5) $$E_{d}=1.6067\times 10^{-6}\cdot L^{3}\cdot m\cdot f_{r}^{2}\cdot \frac{1}{D^{4}}\cdot 2.28$$

where *ρ* is the density of concrete (kg/m^3^), *L* is the length of the specimen (m), *D* is the diameter of the specimen (m), *m* is the mass of the specimen (kg), and *f_r_* is the fundamental frequency of transverse vibration of the specimen (Hz).

The prismatic specimen, due to its sharp corners, tends to induce local stress concentration during freeze–thaw cycling, thereby compromising the accuracy of measurements. In contrast, the cylindrical specimen eliminates this geometric defect and provides a more uniform stress distribution and more stable boundary conditions during freezing. Its circular cross-section facilitates uniform axial restraint at both ends, ensuring that the measured axial stress reliably reflects the actual frost-heave pressure. Therefore, the cylindrical specimen was adopted for this frost-heave stress test.

#### 2.3.4. Ice-Crystal Morphology of Pore Solution

The pore solution used for ice-crystal morphology observation was extracted from freshly mixed cement mortar prepared according to the standard mortar specimen preparation procedure and the mixture proportions listed in Table 4. After mixing, the fresh mortar was immediately transferred into sealed centrifuge tubes and allowed to stand for 30 min. The tubes were then symmetrically placed in a centrifuge and spun at 4000 r/min for 5 min. The supernatant was collected using a rubber dropper and transferred into glass vials to obtain the pore solutions of the three mortar groups [33]. The extracted pore solution was then placed in a custom-built cold-stage microscopic observation system, and the cement pore solution was transferred onto the cold stage using a pipette. Meanwhile, the formation of ice crystals was observed and recorded using a high-resolution digital camera attached to the microscope. When the temperature of the low-temperature cooling stage decreased to the preset freezing temperature, the sample was allowed to continue freezing at that temperature until ice crystals formed. The extracted cement pore solution sample was then maintained at this temperature for 60 s to obtain the final image of the ice crystals [34].

#### 2.3.5. Pore Structure and Ice Content Measurements

Specimens subjected to freeze–thaw cycles and those under standard curing were taken out simultaneously. Cylindrical core samples with dimensions of Φ25 mm × H50 mm were drilled from top to bottom using a coring machine. After extraction, the cylindrical cores were vacuum-saturated with water for 24 h and then subjected to NMR testing [35].

The porosity and pore size distribution were measured under saturated conditions at room temperature. The internal pore structure of the specimens was characterized by determining the transverse relaxation time (*T*_2_) distribution, which reflects the relaxation behavior of hydrogen protons within the pores. Based on the relationship between relaxation time and pore size, the pore structure characteristics of the specimens were further analyzed.

The ice content test was performed under subzero conditions. After water saturation, the specimens were sequentially placed in environments at different preset temperatures (from 0 °C to −20 °C). Taking the signal area at 0 °C as the reference, the relative unfrozen water content at different subzero temperatures was calculated according to Equation (6):

(6) $$S_{\mathrm{water}}=\frac{{\mathrm{FID}}_{T}}{{\mathrm{FID}}_{\mathrm{saturated}}}$$

where S_water_ is the relative unfrozen water content of the specimen during the freeze–thaw process, FID_T_ is the signal intensity of the specimen at a given temperature during the freeze–thaw process, and FID_saturated_ is the signal intensity of the specimen in the fully saturated state at 0 °C.

The relative ice content was calculated according to Equation (7):

(7) $$S_{\mathrm{ice}}=S_{\mathrm{initial}}-S_{\mathrm{water}}$$

where *S*_ice_ is the relative ice content of the specimen during the freeze–thaw process, *S*_initial_ is the initial water content of the specimen before freeze–thaw exposure, taken as 1 in this study, and *S*_water_ is the relative unfrozen water content of the specimen during the freeze–thaw process.

## 3. Results and Discussion

### 3.1. Effect of Freeze–Thaw Cycles on the Macroscopic Properties of Cement Mortar

#### 3.1.1. Evolution of Compressive Strength Under Freeze–Thaw Cycling

**Figure 3:**
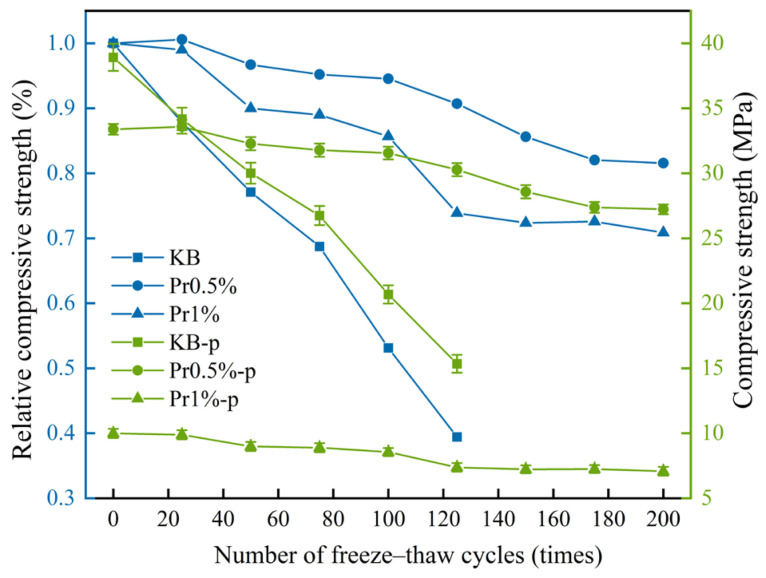
Compressive strength and relative compressive strength of cement mortar subjected to freeze–thaw cycling.

The compressive strength evolution with freeze–thaw cycles is presented in Figure 3. As cycles increased, the compressive strength of the KB-p group declined from 38.92 MPa to 15.35 MPa (a 60.56% reduction), whereas the Pr0.5%-p and Pr1%-p groups decreased from 33.38 MPa to 27.23 MPa (18.42% loss) and from 9.99 MPa to 7.08 MPa (29.13% loss), respectively. Although AFP reduced initial strength (KB > Pr0.5% > Pr1%), the AFP-containing specimens exhibited markedly superior strength retention. The relative compressive strength of the KB-p group dropped to 39.44% after 125 cycles, while the Pr0.5%-p and Pr1%-p groups retained 81.56% and 70.87%, respectively, after 200 cycles.

#### 3.1.2. Evolution of Mass Loss Under Freeze–Thaw Cycling

**Figure 4:**
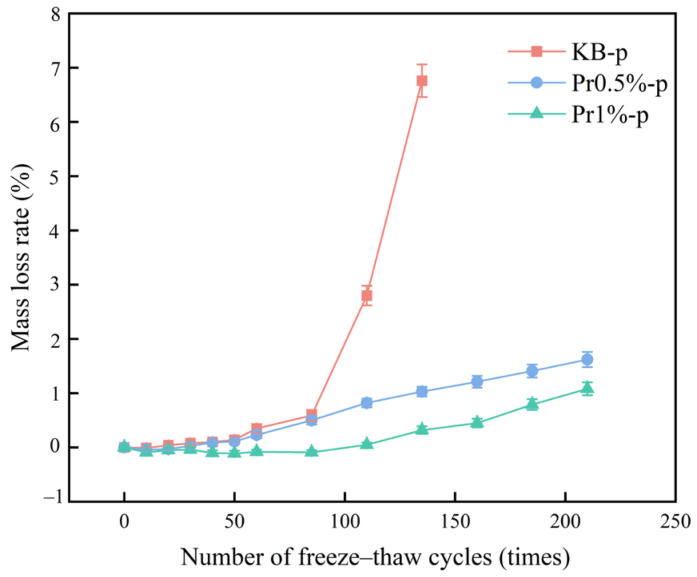
Mass loss ratio of cement mortar subjected to freeze–thaw cycling.

The mass loss ratio evolution with freeze–thaw cycles is shown in Figure 4. All groups exhibited an initial decrease followed by a subsequent increase. The KB-p group reached a mass loss ratio of 6.76% after 135 cycles, exceeding the 5% failure criterion. In contrast, the Pr0.5%-p and Pr1%-p groups exhibited mass loss ratios of only 1.60% and 1.08%, respectively, after 210 cycles. The mass loss ratios ranked as KB > Pr0.5% > Pr1%. These results demonstrate that AFP effectively suppressed mass loss during freeze–thaw cycling, thereby enhancing structural integrity.

### 3.2. Effect of AFP on Freezing Behavior and Pore Structure Evolution

#### 3.2.1. Analysis of Ice-Crystal Morphology of Pore Solution Under Different AFP Dosages

**Figure 5:**
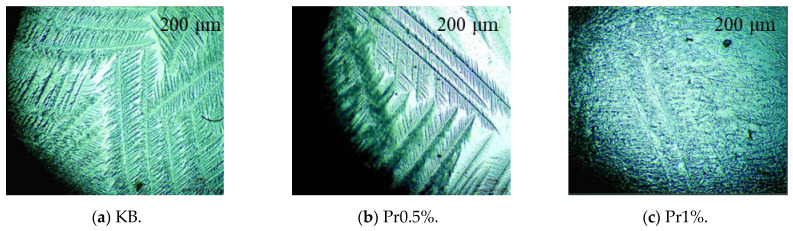
Ice-crystal morphology of pore solutions from cement mortar with varying AFP dosages.

Figure 5 shows the ice-crystal morphologies of pore solutions extracted from cement mortar with varying AFP dosages. In the KB group, ice crystals were predominantly needle-like or elongated with pronounced preferential orientation and well-developed intercrystalline voids, favoring microcrack initiation. With 0.5% AFP, the crystals became shorter, coarser, and more branched. At 1.0% AFP, the morphology further evolved into irregular granular or network-like structures, hindering the formation of large continuous aggregates. These changes indicate that AFP inhibits ordered ice growth and coalescence and modifies the morphology of ice crystals, thereby reducing the risk of localized expansion damage.

#### 3.2.2. Freezing Behavior of Pore Water and Variation in Relative Ice Content Under Different AFP Dosages

**Figure 6:**
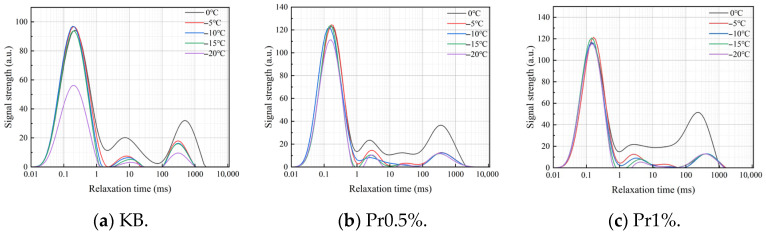
*T*_2_ spectra of cement mortar with different AFP dosages at subzero temperatures.

The *T*_2_ relaxation spectra of cement mortar containing different AFP dosages during cooling are shown in Figure 6. As the temperature decreased, the peak area of the *T*_2_ spectra continuously declined for all groups. When the temperature dropped from 0 °C to −5 °C, the reduction in the *T*_2_ peak area was the most pronounced in each group. After the temperature fell below −5 °C, the *T*_2_ spectral signal of the KB group continued to shift toward lower values, with the third relaxation peak showing the most substantial attenuation. In contrast, the decay rate of the spectral peaks in the AFP-containing specimens was markedly reduced in the subzero temperature range, and the increase in relative ice content became less pronounced within the range of −15 °C to −20 °C.

**Figure 7:**
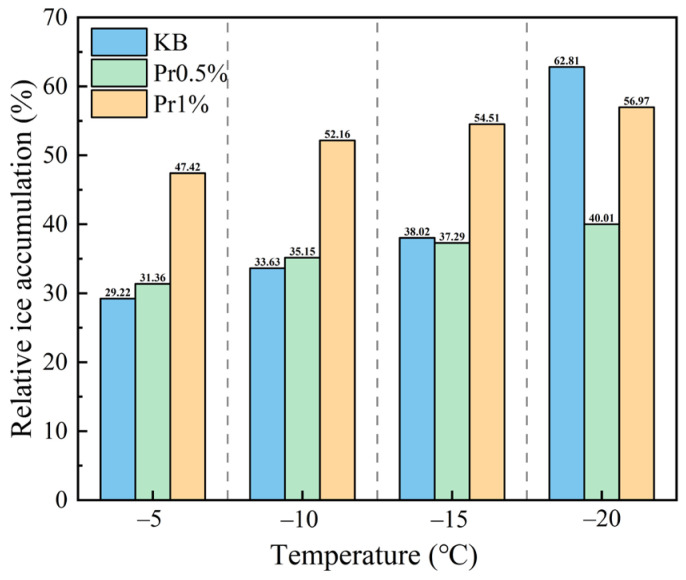
Relationship between relative ice content and temperature.

The calculated relative ice contents are shown in Figure 7. For the KB group, the relative ice contents at −5, −10, −15, and −20 °C were 29.22%, 33.63%, 38.02%, and 62.81%, respectively. The corresponding values for the Pr0.5% group were 31.36%, 35.15%, 37.29%, and 40.01%, respectively, representing a reduction of 22.80% at −20 °C compared with the KB group. For the Pr1% group, the relative ice contents at the corresponding temperatures were 47.42%, 52.16%, 54.51%, and 56.97%, respectively. These values were consistently higher than those of the Pr0.5% group, indicating that the relative ice content did not continue to decrease with increasing AFP dosage.

#### 3.2.3. Evolution of Porosity and Pore Size Distribution Under Freeze–Thaw Cycling

**Figure 8:**
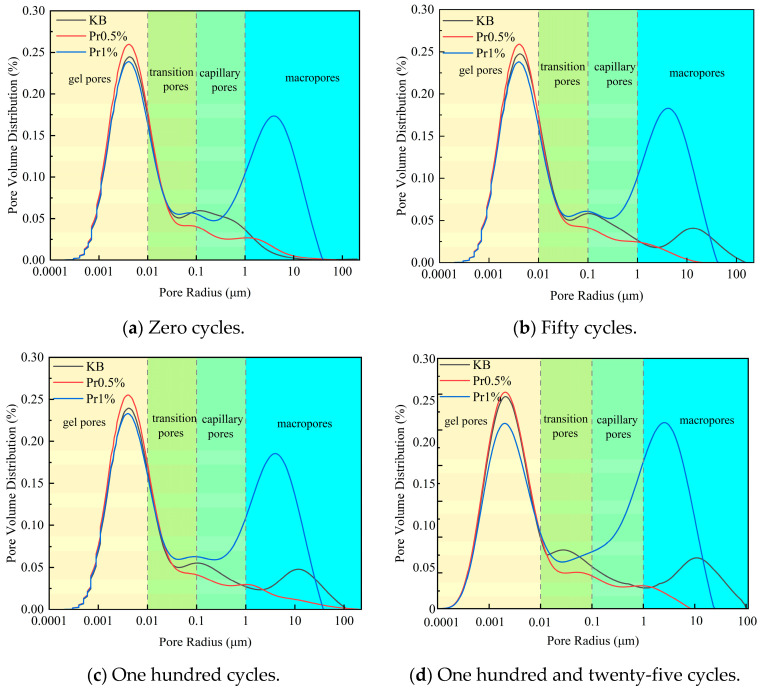
Pore size distribution of cement mortar after different numbers of freeze–thaw cycles.

The pore size distribution of cement mortar with different AFP dosages before and after freeze–thaw cycling is shown in Figure 8. The pore system can be divided into micropores (including gel pores and transition pores, *d* < 0.1 μm), mesopores (capillary pores, 0.1 < *d* < 1 μm), and macropores (*d* > 1 μm). With increasing freeze–thaw cycles, the KB group exhibited a gradual decrease in micropores and a continuous increase in mesopores and macropores, indicating progressive pore coarsening.

In contrast, the AFP-containing specimens showed a relatively slower pore-structure deterioration. Among them, the Pr0.5% group exhibited the most favorable pore refinement, with a comparatively stable micropore proportion and a limited increase in macropores during freeze–thaw cycling. Although the Pr1% group also retarded macropore development to some extent, its overall pore-size distribution did not show a further improvement compared with the Pr0.5% group.

Before freeze–thaw exposure, the pores in the KB group were mainly composed of gel pores and transition pores. After 50 freeze–thaw cycles, the total porosity of the KB, Pr0.5%, and Pr1% groups was 11.25%, 11.55%, and 17.47%, respectively. Compared with the KB group, the total porosity of the Pr0.5% group increased by only 0.30%, whereas that of the Pr1% group increased by 6.22%. From 50 to 125 cycles, the porosity growth of the AFP-containing specimens became relatively gradual, while that of the KB group continued to increase. These results indicate that AFP can retard freeze–thaw-induced pore coarsening, and that the 0.5% dosage provides the most effective stabilization of pore structure.

**Figure 9:**
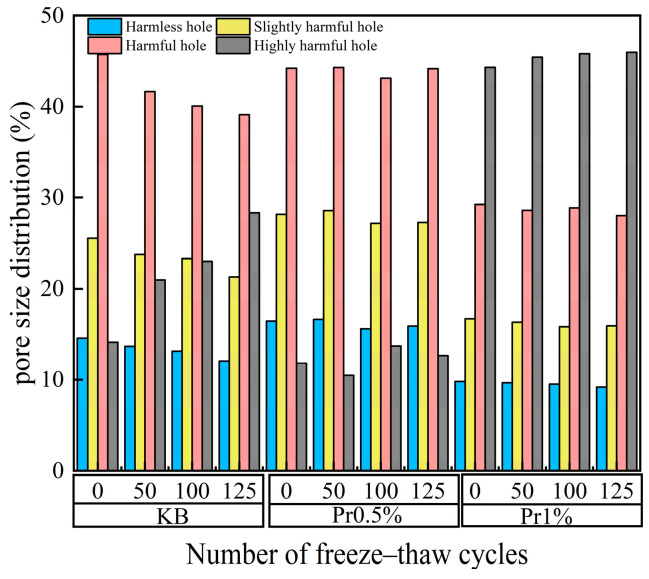
Pore size distribution of saturated cement mortar.

According to the pore hazard classification standard proposed by Wu Zhongwei [36], the quantitative analysis results of the pore structure are shown in Figure 9. During freeze–thaw cycling, the proportions of harmless pores and less harmful pores in the KB group continuously decreased, whereas the proportion of harmful pores increased significantly. After 125 freeze–thaw cycles, the proportion of harmless pores decreased from 14.55% to 12.05%, the proportion of less harmful pores decreased from 25.57% to 21.28%, and the proportion of harmful pores increased from 14.13% to 28.34%.

For the Pr0.5% group, the initial proportions of harmless pores and less harmful pores were 16.43% and 28.17%, respectively, both higher than those of the KB group, while the proportion of harmful pores was 11.81%. After 125 freeze–thaw cycles, the proportion of harmless pores remained at 15.88%, the proportion of less harmful pores decreased to 27.28%, and the proportion of harmful pores increased to 12.65%.

For the Pr1% group, the initial proportions of harmless pores, less harmful pores, and harmful pores were 9.80%, 16.69%, and 29.24%, respectively, while the proportion of highly harmful pores reached 44.33%. After 125 freeze–thaw cycles, the proportion of highly harmful pores further increased to 45.99%, whereas the proportions of harmless pores and less harmful pores decreased to 9.17% and 15.91%, respectively.

These results indicate that, under freeze–thaw cycling, the pore structure of mortar generally tends to shift from a fine-pore-dominated system to a coarse-pore-dominated one. The incorporation of AFP can retard this process to a certain extent, and the 0.5% AFP dosage exhibits the best performance in suppressing the increase in total porosity, maintaining the proportion of fine pores, and limiting the development of highly harmful pores.

### 3.3. Evolution of Frost-Heaving Strain and Frost-Heaving Stress of Cement Mortar with Different AFP Dosages

#### 3.3.1. Experimental Results of Frost-Heaving Deformation and Stress

**Figure 10:**
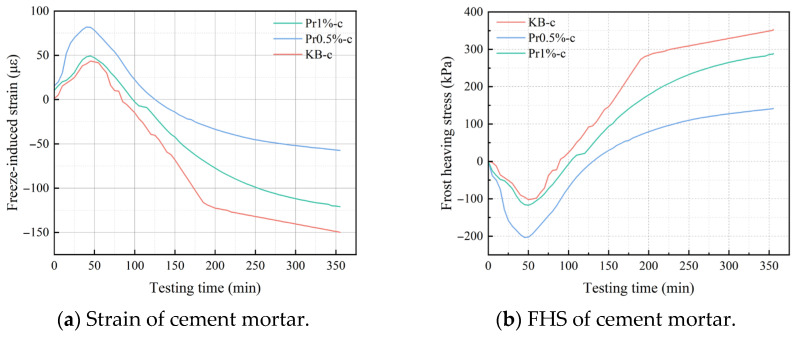
Evolution curves of strain and frost-heaving stress of cement mortar with different AFP dosages during freezing at −20 °C.

Figure 10 shows the evolution curves of strain and frost-heaving stress of cement mortar containing different AFP dosages during the freezing process at −20 °C. In the initial stage of freezing, the strain of all specimens gradually increased, with the response being dominated by thermal contraction during cooling. After approximately 45 min, the strain reached a peak and then began to decrease. The period from 45 to 210 min corresponded to the main stage of intensive pore-water freezing, during which significant volumetric effects were induced; accordingly, the frost-heaving stress increased rapidly in the same stage. The KB-c group exhibited the highest frost-heaving stress throughout the entire freezing process. After the incorporation of AFP, the frost-heaving stress decreased markedly, with the lowest value observed in the Pr0.5%-c group, followed by the Pr1%-c group, and both were significantly lower than that of the KB-c group.

**Figure 11:**
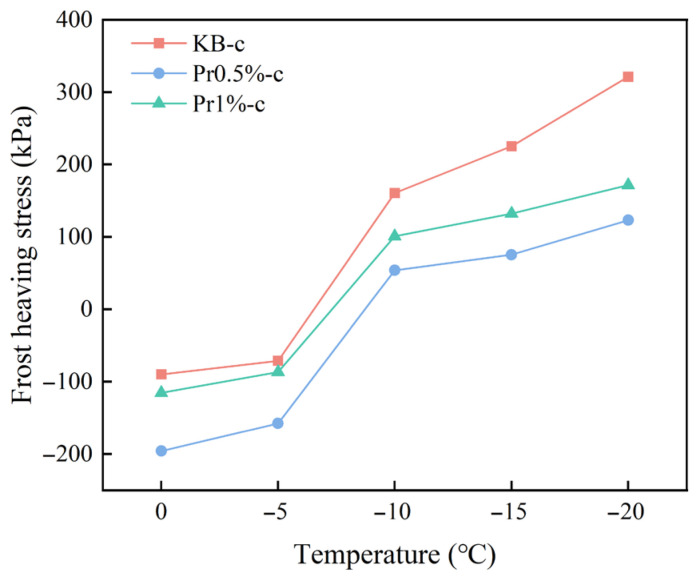
Relationship between temperature and frost-heaving stress.

The relationship between frost-heaving stress and temperature under subzero conditions for different specimens is shown in Figure 11. In the range of 0 °C to −5 °C, the frost-heaving stress in all groups remained negative, indicating a state of contraction stress. As the temperature further decreased, the frost-heaving stress increased rapidly. In the KB-c group, the frost-heaving stress reached 160.57 kPa at −10 °C and further increased to 321.15 kPa at −20 °C. For the Pr0.5%-c and Pr1%-c groups, the frost-heaving stresses at −20 °C were 123.04 kPa and 171.29 kPa, respectively. Among them, the Pr0.5% group exhibited the lowest stress level and the most gradual increase during cooling.

These results indicate that the incorporation of AFP can effectively reduce the internal frost-heaving stress of cement mortar during freezing, thereby mitigating the expansive damage induced by the water-ice phase transition to the material skeleton. This effect is most pronounced at an AFP dosage of 0.5%.

#### 3.3.2. Model Assumptions and Derivation

To elucidate the mechanism by which AFP influences frost-heaving stress in cement mortar, a frost-heaving stress model was developed based on thermodynamic principles and fractal geometry. Rather than serving as a fully generalized predictive tool, the model was intended to provide a mechanistic framework linking relative ice content, pore-scale freezing behavior, ice-crystal boundary complexity, and macroscopic frost-heaving stress. In this framework, relative ice content reflects the effective amount of frozen water, the critical pore radius defines the temperature-dependent freezing condition within the pore system, and the fractal dimension describes the geometric complexity of the ice-crystal boundary. Thus, frost-heaving stress evolution can be interpreted as the coupled result of ice formation, pore confinement, and stress transfer in the cement mortar matrix. The model assumptions and derivation are described below:

(1) The pore system in cement mortar exhibits a continuous pore size distribution, and the freezing of pore water during cooling proceeds progressively from larger pores to smaller pores.

(2) The boundaries of ice crystals formed within the pores possess fractal characteristics, and the complexity of the interface can be characterized by the fractal dimension *D_f_*.

(3) At a given temperature, the ice–water two-phase system in the frozen pores is in a state of local thermodynamic equilibrium.

(4) When no significant damage has yet occurred in the matrix during the initial stage of freezing and thawing, the cementitious matrix can be approximately treated as a linear elastic body.

(5) The macroscopic frost-heaving stress originates from the combined effect of microscopic crystallization pressures generated in a large number of frozen pores, and the transformation from the microscopic scale to the macroscopic scale can be described by an equivalent stress transfer coefficient.

Based on the above assumptions, the freezing of pore water in cement mortar is constrained by pore size and therefore exhibits a typical confined-freezing behavior. According to the Gibbs–Thomson effect [37], a smaller pore radius leads to a larger curvature of the ice–water interface and a lower equilibrium freezing temperature. At a given temperature *T*, only pores with a radius greater than the critical freezing radius *r_c_* can undergo freezing, as given in Equation (11).

(8) $$r_{c}(T)=\frac{2\sigma_{i}T_{m}}{\rho_{i}L_{m}}\cdot \frac{1}{T_{m}-T_{0}}$$

where *T*_0_ is the freezing temperature of free water (°C); *T*_m_ is the actual freezing temperature of pore water (°C); *σ_i_* is the ice–water interfacial energy, taken as 0.0409 J/m^2^ in this study; *ρ_i_* is the density of ice; *Lm* is the latent heat of fusion of ice; and *r* is the effective pore radius. When the pore radius satisfies *r* > *r_c_*, the pore water is thermodynamically capable of freezing.

When pore water undergoes a liquid–solid phase transition, ice crystals grow under the confinement of the pore walls and exert crystallization pressure on the pore walls. Based on the Clausius–Clapeyron phase equilibrium relation [38], the relationship between crystallization pressure and phase-transition temperature can be expressed by Equation (9). By integrating this relation over the interval from *T*_0_ to *T*_m_, a quantitative relationship between ice-crystal growth pressure and the freezing-point depression can be established, as shown in Equation (10). Furthermore, by substituting the Gibbs–Thomson relation into this expression, the correlation between ice-crystal growth pressure and pore scale can be obtained, as given in Equation (11).

(9) $$\frac{dP}{dT}=\frac{L_{m}}{T(v_{\mathrm{liquid}}-v_{\mathrm{ice}})}$$

(10) $$P_{\mathrm{ice}}=\frac{L_{m}\Delta T}{T_{0}(V_{\mathrm{ice}}-V_{\mathrm{liquid}})}$$

(11) $$P_{c}=\frac{2\sigma_{i}T_{0}}{\rho_{i}L_{m}r_{c}}\cdot \frac{L_{m}\rho_{i}}{T_{0}}=\frac{2\sigma_{i}}{r_{c}}$$

where d*P*/d*T* denotes the rate of change in phase-transition pressure with temperature; *T* is the phase-transition temperature (°C); *V*_liquid_ is the specific volume of liquid water (m^3^/kg); and *V*_ice_ is the specific volume of ice (m^3^/kg).

Considering that the low-temperature microscopic images show that the ice-crystal boundaries within the pores are highly irregular, the conventional assumption of a smooth interface is insufficient to accurately describe the actual stress state. Therefore, the fractal dimension of the ice-crystal boundary, *D_f_*, was introduced to modify the crystallization pressure. For a fractal interface, the effective interfacial area *A_f_* satisfies the scaling relationship with the characteristic scale *r*, as shown in Equation (12). After normalization by introducing the maximum characteristic scale *r*_max_, Equation (13) can be obtained. Accordingly, the modified effective crystallization pressure can be expressed by Equation (14). During cooling, the freezing behavior is governed by the critical pore radius *r_c_*. By substituting *r* = *r_c_*, the expression for the effective crystallization pressure considering the fractal morphology of ice crystals can be obtained, as shown in Equation (15).

(12) $$A_{f}(r)\propto r^{D_{f}}$$

(13) $$\frac{A_{f}(r)}{A_{f}(r_{\max})}={\left(\frac{r}{r_{\max}}\right)}^{D_{f}-1}$$

(14) $$P=\frac{2\sigma_{i}}{r}\cdot {\left(\frac{r}{r_{\max}}\right)}^{D_{f}-1}$$

(15) $$P_{c}(T)=\frac{2\sigma_{i}}{r_{c}(T)}\cdot {\left(\frac{r_{c}(T)}{r_{\max}}\right)}^{D_{f}-1}$$

The macroscopic frost-heaving stress arises from the combined effect of microscopic crystallization pressures generated within a large number of frozen pores. Assuming that the microscopic pressure is uniformly distributed within the material and effectively transferred through the matrix, the macroscopic frost-heaving stress can be expressed by Equation (16), in which *ϕ_ice_* is the relative ice content measured by NMR and *η* is the stress transfer coefficient. By using Equation (5), the stress transfer coefficient at each temperature point can be determined, as given in Equation (17), and the average stress transfer coefficient can then be obtained by statistical averaging over multiple temperature points, as shown in Equation (18). Finally, a macroscopic frost-heaving stress model incorporating the fractal characteristics of ice crystals is established, as expressed in Equation (19).

(16) $$\sigma =\eta \phi_{ice}P_{c}(T)$$

(17) $$\eta ’=\frac{E_{d}\varepsilon }{\phi_{ice}P_{c}(T)}$$

(18) $$\eta =\frac{1}{N}\sum_{i=1}^{N}\eta ’(T_{i})$$

(19) $$\sigma_{f}(T)=\eta \phi_{\mathrm{ice}}(T)P_{c}(T){\left(\frac{r_{c}(T)}{r_{\max}}\right)}^{D_{f}-1}$$

where *ϕ_ice_* is the relative ice content, which can be obtained from the NMR measurements, and *η* is the stress transfer coefficient, reflecting the efficiency of the transformation from microscopic pressure to macroscopic stress.

The core parameters of the model were calibrated based on the experimental results. The fractal dimension of ice-crystal morphology, *D_f_*, was determined from the ice-crystal contours extracted from low-temperature microscopic images using the box-counting method. Prior to analysis, the images were subjected to grayscale conversion and background subtraction in ImageJ software (version 1.54p, National Institutes of Health, Bethesda, MD, USA). The calculated *D_f_* values for the KB, Pr0.5%, and Pr1% groups were 1.79, 1.84, and 1.85, respectively. The relative ice content, *ϕ_ice_*, was derived from the measured values obtained in the subzero NMR tests presented in Section 3.2.2 over the temperature range from 0 °C to −20 °C. The critical pore radius, *r_c_*, was determined based on the Gibbs–Thomson effect in conjunction with the NMR-derived pore size distribution data; the corresponding critical freezing pore radius at different temperatures are listed in Table 5. The stress transfer coefficient, *η*, was back-calculated from the measured frost-heaving stress values at three characteristic temperatures—namely −10 °C, −15 °C, and −20 °C—and subsequently averaged. The resulting values are presented in Table 6.

#### 3.3.3. Theoretical Calculation of Frost-Heaving Stress

**Figure 12:**
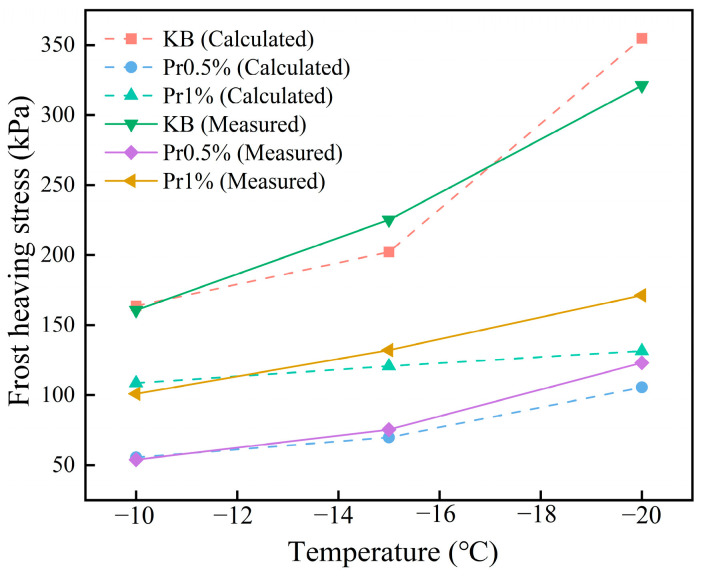
Comparison of measured and model-predicted frost-heaving stress as a function of temperature for cement mortar with different AFP dosages.

Within the continuous cooling interval from −10 °C to −20 °C, a comparative analysis was conducted between the model-calculated frost-heaving stress and the measured values for each cement mortar group, as shown in Figure 12. As the temperature continuously decreased, both the measured and model-predicted frost-heaving stresses exhibited a gradual increasing trend. At −20 °C the theoretical frost-heaving stresses of the KB, Pr0.5%, and Pr1% groups were 354.85 kPa, 105.51 kPa, and 131.50 kPa, respectively. Comparison between the calculated and measured results showed that all groups followed the same overall trend, with the KB group exhibiting the highest stress, the Pr0.5% group the lowest, and the Pr1% group lying in between.

The overall average deviation between the theoretical predictions and the measured values was approximately 9.63%. The measured frost-heave stress only shows that AFP reduces the final stress level, and the model further reveals the root cause of this reduction: the joint effect of the reduction in effective ice formation, the weakening of stress transfer and the increase in ice-crystal boundary complexity. For the pr0.5% group, the lower relative ice content and the smaller stress transfer coefficient lead to the lowest calculated and measured values of frost-heave stress. For pr1.0% group, although AFP still changed the ice-crystal morphology, its high relative ice content and poor pore structure weakened the effect of stress reduction. This also explains why increasing the dosage of AFP from 0.5% to 1.0% cannot further improve the freeze–thaw durability.

Therefore, the model can not only effectively explain the internal relationship between relative ice content, stress transfer, ice-crystal morphology evolution and frost-heave stress response, but also provide important theoretical support for understanding the frost resistance mechanism of AFP in cement mortar. However, it should be pointed out that the current model is more suitable for the analysis of frost-heave stress evolution trend, and its applicability and quantitative accuracy still need to be verified under a wider range of experimental conditions.

#### 3.3.4. Sensitivity Analysis of Model Parameters and the Influence of AFP on Frost-Heaving Stress

Based on the frost-heaving stress model established above, the effect of AFP dosage on frost-heaving stress cannot be evaluated solely from the macroscopic experimental results. It should also be analyzed in conjunction with the variations in the key model parameters, including the stress transfer coefficient *η*, the relative ice content *ϕ_ice_*(*T*), and the fractal dimension of the ice-crystal boundary, *D_f_*.

**Figure 13:**
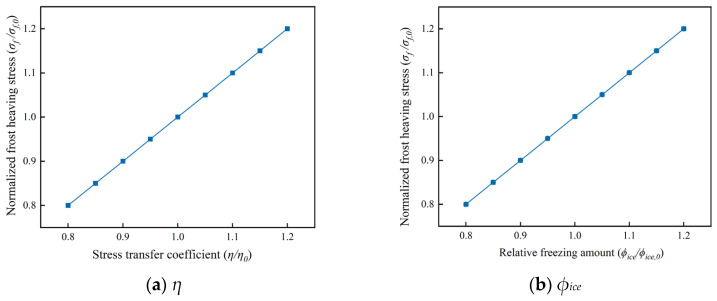
Theoretical effects of *η* and *ϕ_ice_* on normalized frost-heaving stress.

The sensitivity analysis of the model shows that both η and *ϕ_ice_*(*T*) are first-order controlling factors of frost-heaving stress. In the theoretical expression, they have the same linear weighting, and their logarithmic sensitivities are both equal to 1. As jointly indicated by Table 7 and Figure 13, when either *η* or *ϕ_ice_*(*T*) varies independently, the normalized frost-heaving stress exhibits a linear response: for every 1% change in *η* or *ϕ_ice_*(*T*), the theoretical frost-heaving stress changes correspondingly by 1%. When *η* or *ϕ_ice_*(*T*) is reduced by 10% individually, the stress decreases by 10%; when both are reduced simultaneously by 10%, the stress decreases by approximately 19%. This indicates that reducing the effective ice-forming action and lowering the stress transfer efficiency are equally important for suppressing frost-heaving stress. Among them, the variation in *η* directly reflects the extent to which AFP weakens the transmission pathway of freezing-induced expansion, whereas the variation in *ϕ_ice_*(*T*) corresponds to the increase or decrease in effective ice formation. Therefore, at relatively low AFP dosages, the reduction in frost-heaving stress is mainly attributable to the decrease *in ϕ_ice_*(*T*).

**Figure 14:**
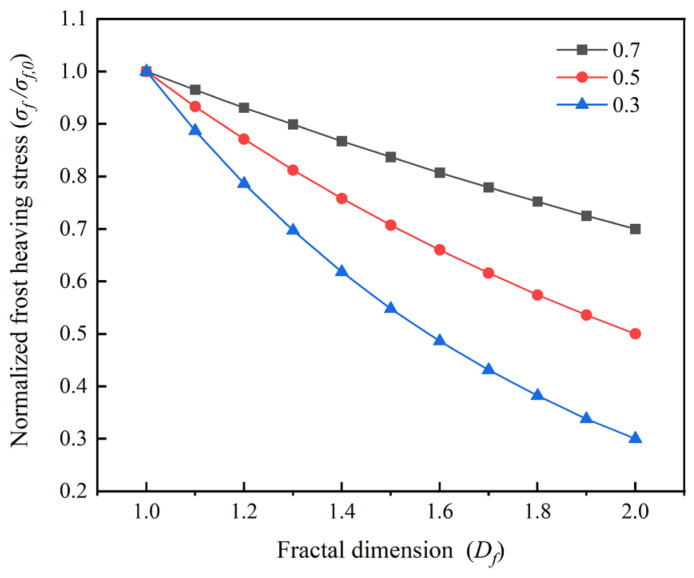
Influence of *D_f_* on normalized frost-heaving stress under different *r_c_*/*r_max_* conditions.

An increase in *D_f_* indicates that the ice-crystal boundary becomes more complex and irregular, the regular and continuous growth of ice crystals is suppressed, local stress concentration is weakened, and the overall frost-heaving stress is reduced. Figure 14 shows that, under different pore-constraint conditions, the curves describing the effect of *D_f_* on normalized frost-heaving stress all exhibit a decreasing trend, although the magnitude of the reduction differs significantly. The model calculations indicate that for every 0.1 increase in *D_f_*, the frost-heaving stress decreases by approximately 3.5% when r_c_/r_max,_ by 6.7% when r_c_/*r*_max_ = 0.5, and by as much as 11.3% when *r*_c_/*r*_max_ = 0.3. These results suggest that the effect of *D_f_* on frost-heaving stress is strongly coupled with the pore-constraint condition: the stronger the confinement, the greater the contribution of ice-crystal morphology regulation to stress release.

### 3.4. Mechanistic Discussion of AFP-Enhanced Frost Resistance in Cement Mortar

**Figure 15:**
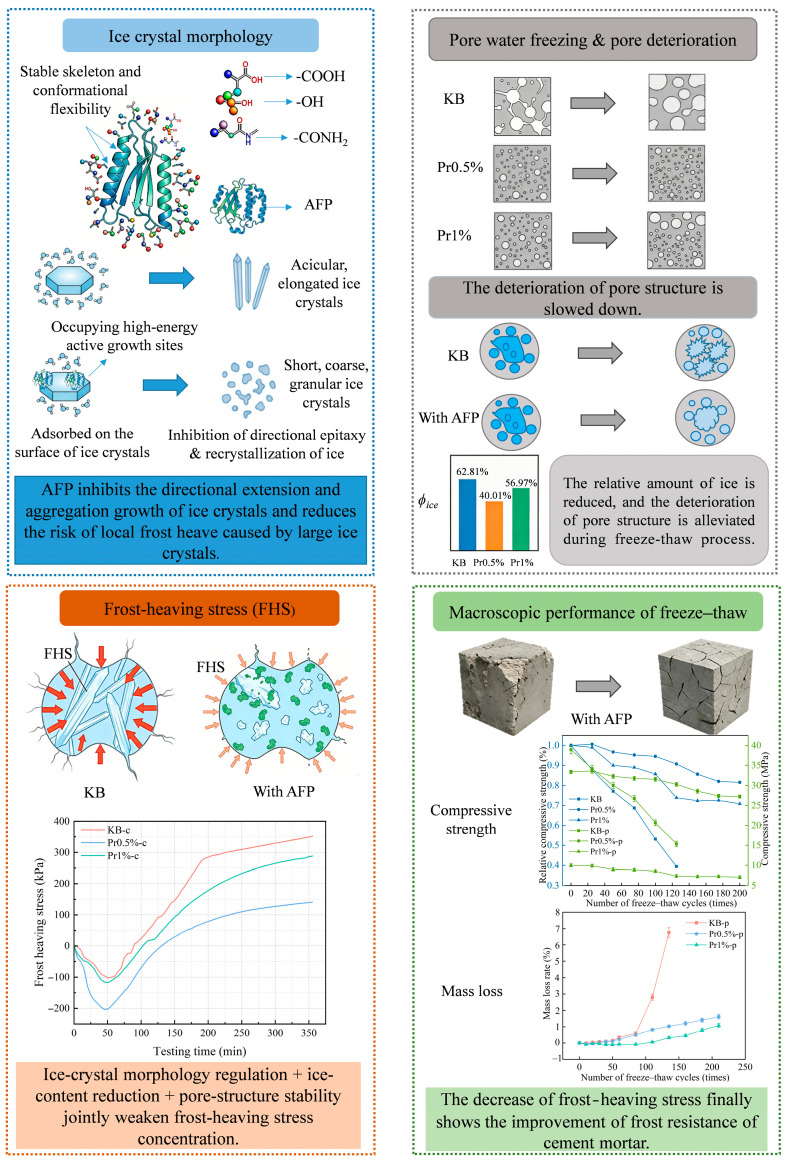
Schematic of AFP effects on freezing behavior and freeze–thaw durability of cement mortar.

Through a macro–micro multiscale measurement approach, this study demonstrates that the improvement in the frost resistance of cement mortar by AFP is mainly manifested in four aspects: regulation of ice-crystal growth morphology, reduction in the relative degree of freezing, retardation of pore-structure coarsening, and suppression of frost-heaving stress accumulation. Among the investigated dosages, 0.5% AFP exhibited the best overall frost-resistance performance. A schematic illustration of the underlying mechanism is presented in Figure 15.

The high proportion of polar and charged amino acid residues in AFP provides the structural basis for its adsorption at the ice–water interface [39,40], while the relatively high contents of cystine and proline contribute to the stability of its molecular conformation [41]. Polar groups carried by these residues, including -OH, -COOH, -C=O, -CONH_2_, and -NH_3_^+^, become enriched at the interface and occupy growth steps, edges [42], and defect sites through hydrogen bonding and electrostatic interactions. This impedes the orderly deposition of water molecules and weakens the directional growth of ice crystals. Partial oriented dendritic growth was still observed in the Pr0.5% group, whereas the preferential orientation was more evidently disrupted in the Pr1% group, leading to a more irregular and network-like morphology. [43]. The NMR results further show that AFP, especially at a dosage of 0.5%, retards pore coarsening during freeze–thaw cycling and preserves a more favorable pore-size distribution. In particular, after 125 cycles, the proportion of highly harmful pores decreased from 28.34% in the KB group to 12.65% in the Pr0.5% group. This improved pore structure is closely associated with the reduction in effective ice formation and frost-heaving stress. At −20 °C, the relative ice contents of the KB, Pr0.5%, and Pr1% groups were 62.81%, 40.01%, and 56.97%, respectively, while the corresponding frost-heaving stresses were 321.15, 123.04, and 171.29 kPa. These results indicate that AFP not only reduced the effective degree of ice formation, but also suppressed the generation and transmission of stress during freezing. Ultimately, these effects were manifested in substantially higher compressive strength retention and a markedly lower mass loss rate after freeze–thaw cycling, indicating an overall enhancement of macroscopic performance.

It is worth noting that the effect of AFP on the performance of cement mortar did not continue to improve with increasing dosage. Compared with the KB group, the increases in porosity for the Pr0.5% and Pr1% groups were 0.32% and 6.23%, respectively, while the corresponding reductions in frost-heaving stress were 61.69% and 46.66%. Although the 1% group still exhibited a certain capacity to regulate ice-crystal growth, its initial porosity increased from 11.22% to 17.45%, and its initial compressive strength decreased from 38.90 MPa to 9.99 MPa. This indicates that, under the conditions of the present study, a relatively high AFP dosage may have adversely affected the early-stage structural formation and compactness of the cement matrix. Therefore, the influence of AFP on cement mortar performance can be understood as the result of a balance between antifreeze benefits and structural costs.

Overall, the strengthening mechanism of AFP in cement mortar should be interpreted as the joint effect of pore structure stability and ice-crystal morphology, rather than only affected by the control of ice-crystal growth. However, when the dosage is further increased, although AFP may still exert a certain influence on the freezing behavior, the adverse effects associated with the deterioration of the initial microstructure may offset the overall performance gains. Therefore, within the scope of this study, an AFP dosage of 0.5% exhibited the most favorable overall frost-resistance performance.

## 4. Conclusions

This study investigated the effect of soybean antifreeze protein (AFP) on the freeze–thaw durability of ordinary Portland cement mortar. Through macroscopic performance tests, low-temperature microscopic observation, NMR-based pore-structure characterization, frost-heaving stress monitoring, and theoretical model analysis, the mechanism by which AFP influences the freezing behavior of pore solution and suppresses frost-heaving damage was revealed. Based on the above experimental and analytical results, the main conclusions are as follows:

(1) AFP incorporation improved the freeze–thaw durability of cement mortar, with the 0.5% dosage outperforming the 1.0% dosage. After 200 cycles, the relative compressive strengths of the Pr0.5% and Pr1% groups remained at 81.56% and 70.87%, respectively. The mass loss ratio of the KB group reached 6.76% after 135 cycles, whereas that of the Pr0.5% group was only 1.60% after 210 cycles.

(2) AFP transformed ice crystals from needle-like into fine granular forms, reducing the relative ice content and mitigating pore coarsening during freeze–thaw cycling. At −20 °C, the relative ice contents of the KB, Pr0.5%, and Pr1% groups were 62.81%, 40.01%, and 56.97%, respectively, while the corresponding frost-heaving stresses were 321.15, 123.04, and 171.29 kPa. After 125 cycles, the proportion of highly harmful pores in the KB group reached 28.34%, compared with only 12.65% in the Pr0.5% group.

(3) The theoretical model showed good agreement with experimental results, with an overall average deviation of approximately 9.63%. The predicted frost-heaving stresses were consistent with the measured values in both trends and group rankings, indicating that AFP’s mechanism for improving freeze–thaw durability mainly involves reducing effective ice formation, weakening stress transfer, and increasing ice-crystal boundary complexity.

(4) The improvement of freeze–thaw properties of cement mortar by AFP was not further enhanced with the increase in dosage from 0.5% to 1.0%. Although the pr1.0% group still showed a certain ability to control the growth of ice crystals, its initial porosity increased from 11.22% to 17.45%, and its initial compressive strength decreased from 38.90 MPa to 9.99 MPa, indicating that higher dosage of AFP may have an adverse effect on the early structure formation and compactness of the cement matrix, thereby weakening its improvement effect on the overall freeze–thaw durability.

In summary, soybean antifreeze protein, as a biomass-derived functional admixture, exhibits clear potential for improving the freeze–thaw durability of cement mortar. Within the scope of this study, an AFP dosage of 0.5% showed the most favorable overall performance, whereas a further increase to 1.0% led to structural costs that partially offset the antifreeze benefits. These findings may provide a useful reference for the development of green antifreeze modification strategies for cement-based materials in cold regions.

## Figures and Tables

**Table 1:** Physical properties of cement.

Fineness (%)	Water Require (%)	Specific Surface Area (m^2^/kg)	Setti Time (min)		Flexural Strength (MPa)			Compressive Strength (MPa)		
			Initial	Final	3 d	7 d	28 d	3 d	7 d	28 d
0.6	28.5	388	172	223	4.9	6.1	7.9	0.6	28.5	45.7

**Table 2:** Chemical composition of cement.

CaO	SiO_2_	Fe_2_O_3_	Al_2_O_3_	MgO	Na_2_Oeq	Others	LOI (%)	Soluble Residue (%)
55.28	25.45	4.58	12.5	2.23	0.72	0.14	2	1.0

**Table 3:** Amino acid composition of AFP.

Amino Acid	Content (mg/g Protein)	Relative Content (%)
Aspartic acid	3.47	6.89
Threonine	2.03	4.03
Serine	2.68	5.32
Glutamic acid	6.16	12.23
Glycine	4.88	9.69
Alanine	1.00	1.99
Cystine	6.97	13.84
Valine	2.23	4.43
Methionine	0.57	1.13
Isoleucine	2.11	4.19
Leucine	1.52	3.02
Tyrosine	2.82	5.60
Phenylalanine	1.57	3.12
Histidine	1.45	2.88
Lysine	2.24	4.45
Arginine	2.39	4.74
Proline	6.29	12.49
Total	50.37	100.00

**Table 4:** Compositions of cement mortar specimens used for different tests.

Specimen Type	Specimen ID	w/b	Cement (g)	Standard Sand (g)	AFP Mass (g)	Water (g)	Test Purpose
Prismatic	KB-p	0.50	450	1350	0.00	225	Freeze–thaw, strength, mass loss, NMR
Prismatic	Pr0.5%-p	0.50	450	1350	2.25	225	Freeze–thaw, strength, mass loss, NMR
Prismatic	Pr1%-p	0.50	450	1350	4.50	225	Freeze–thaw, strength, mass loss, NMR
Cylindrical	KB-c	0.50	900	2700	0.00	450	Frost-heaving stress monitoring
Cylindrical	Pr0.5%-c	0.50	900	2700	4.50	450	Frost-heaving stress monitoring
Cylindrical	Pr1%-c	0.50	900	2700	9.00	450	Frost-heaving stress monitoring

**Table 5:** Critical freezing pore radius at different temperatures.

Temperature (°C)	−1	−5	−10	−15	−20
frozen pores (μm)	0.0682	0.0136	0.0068	0.0045	0.0034

**Table 6:** Stress transfer coefficient at different AFP dosages.

AFP Content (%)	0	0.5	1
*η*	137.54	26.38	49.88

**Table 7:** Effect of model parameter variations on normalized frost-heaving stress.

Parameter Variation	Normalized Frost-Heaving Stress	Theoretical Reduction
*η* decreased by 10%	0.900	10%
*ϕ_ice_*(*T*) decreased by 10%	0.900	10%
*η* and *ϕ_ice_*(*T*) decreased by 10%	0.810	19%
*D_f_* increased by 0.1, *r_c_*/*r_max_* = 0.7	0.965	3.5%
*D_f_* increased by 0.1, *r_c_*/*r_max_* = 0.5	0.933	6.7%

## Data Availability

The original contributions presented in this study are included in the article. Further inquiries can be directed to the corresponding author.
